# Report of the Commissioners Appointed to Take the Census of Ireland for the Year 1841. Presented to Both Houses of Parliament by Command of Her Majesty

**Published:** 1844-07

**Authors:** 


### Art. II.

*Report of the Commissioners appointed to take the Census of Ireland for the year* 1841. *Presented to both Houses of Parliament by Command of Her Majesty.—Dublin*, 1843. Folio.

It is not our intention to present an analysis of all the matters comprised in this very valuable and voluminous statistical survey of Ireland, comprising ample and (excepting where circumstances precluded perfect accuracy,) precise information on the domestic, social, and sanitary condition of her population. We find, however, so much medical and historico-medical in formation in the 'Report on the Tables of Death,' from the pen of Mr. Wilde, which it comprises, that we consider that we should be failing in our duty as journalists did we not furnish some view of it to our readers.

The materials whence his remarks are derived are ample, dealing as Mr. Wilde does with the mortality of Ireland for the ten years ending on the 6th of June, 1841—a mortality of about 1,187,374, and probably even exceeding this.

Of this general mortality, 911,619 occurred in the open country, including minor towns and villages; 234,173 deaths took place in towns; and 41,582 deaths in the different hospitals and sanitary institutions. The proportionate deaths in the two sexes were 100 males to 91·24 females in the general mortality, with some variations in these proportions in the different districts.

We are assured, however, that the 1,187,374 are not all the deaths that occurred in Ireland during the ten years specified; and it can only be conjectured what proportion of the total decennial mortality they constitute, for there existed no proper annual registration of the kingdom.

They appear, according to Mr. Wilde's opinion to be *minus* about one fourth. He remarks that, taking an average population between the census of 1831 and 1841, and the average of the entire deaths returned upon the present inquiry for that period, we find the annual proportion of deaths to be 1·48 per cent., or one death in every 67·13 (we would say 67·56,—REV.) of the population. Supposing, then, the actual annual mortality in Ireland to be 2 per cent., or 1 in 50, in round numbers, then these deaths are, as a whole, *minus* about one fourth. The statement of the census commissioners on this inaccuracy show it to be very considerable indeed. In 1834, in a population of 7,943,940, the mortality returned was 26,623, being 1 to 82·2 or 1·22 per cent. There can be no doubt that this is very incorrect; and the inaccuracy becomes very glaring indeed when we find that in 1839, in a population of 8,092,947, the deaths were 140,239, or 1 in 57·7, or, in other words, 1·73 per cent.; and that in 1840, the population being 8,133,934, the mortality was 141,536, or 1 in 57·5, or 1·74 per cent. The commissioners propose the same corrections of the inaccuracy as Mr. Wilde, and on the following grounds;

"In propositions founded on numbers so imperfect, we may take the last as the most correct. Still, as the errors of voluntary returns are naturally corrected by being spread over a number of years, it is probable that the most satisfactory result will be obtained by using the average population and average deaths of the years from 1836 to 1840, which we believe to be of very equal value; thus: 1839, population 8,052,170, deaths 135,359, average mortality 1 in 59·5, or 1·68 per cent.; and as there can be no reason for supposing the mortality less than 1 to 45—on the contrary, many circumstances would appear to place it higher—we are led to apprehend a deficiency in the death returns amounting to about one fourth.

"If we apply this correction to the total number of deaths, we shall have the following data for computation:

| | | |
|---|---|---|
| Population of 1831 . . . | 7,767,407 | |
| Deduct the army and followers then in Ireland . . . . | 29,486 | |
| | | 7,737,915 |
| Add births between 1831 and 1841 . . . | | 2,496,826 |
| | | 10,234741 |
| Deduct deaths per table . . | 1,187,374 | |
| Ditto, increased by a fourth . . | 296,843 | |
| | | 1,484,217 |
| Total . . . . . . | | 8,750,524 |

"This total would be the computed population for 1841, and the difference between it and that given by enumeration—viz. 575,400—would remain to be accounted for by emigration or other causes. This number will be found to agree very nearly with that taken as the amount of the subtraction from the population between 1831 and 1841, at which we arrived by independent and totally different means.

"These calculations, however, interesting as they may be, are inserted here, not so much for their own value (not being based on numbers positively ascertained,) as to show how deficient we are in the data requisite for the correct deduction of the value of life assurances and similar subjects in Ireland, which recent information and better materials in other countries, more especially in England, have shown to vary in different circumstances and conditions of life, to an extent wholly unknown before." (Census Commissioners' Report, p. xliv.)

A sketch of the history of Irish medicine, which will be read with interest, serves as a preface to Mr. Wilde's nosological table and classification of the enormous mass of materials he has to deal with.

"Herb-doctors," it seems, existed in Ireland during the last and beginning of the present century, and some of them acquired reputation even in the metropolis. These persons acquired their information from Irish medical MS. productions of the middle ages, many of which are still extant in the Royal Irish Academy and the King's Inns Library, Dublin. Mr. Wilde enumerates and describes several of them. They bear a considerable resemblance to the works of those persons who, in various parts of Europe, have been called *Arabistæ*, men who in the middle ages occupied themselves with translating, commonly into Latin, but sometimes into the vernacular of their country, and commenting upon the writings of the Arabians, such as Mesue, Haly Abbas, and Avicenna, and whose knowledge of Greek and Roman medicine reached them through Arabian channels. Their works, or considerable portions of them, are to be found in the collections of Luisinus and Gruner, especially of the latter. England owned some of them, as the 'Rosa Anglica' of John of Gaddesden, (Professor of Merton College, Oxford,) written about 1305, proves. Such works seem to have abounded in Ireland, showing the love of letters for which her highly-gifted population has ever been distinguished; for though many are mentioned by Mr. Wilde, he expressly states that he has specified only those from which he has derived his Irish nosology, (so valuable and interesting a feature in his Report,) but he has not given a catalogue of any portion of Irish medical literature—thus indicating the great amount of the whole stock. We consider the circumstance of the Irish herb-doctors deriving their information from the medical literature of a *bygone* day confirmatory of an opinion we have ever entertained, that popular medicine is the cast-clothes of the profession; the public move in the same track as ourselves, but at a great distance behind. Mr. Wilde says:

"During the fourteenth and fifteenth centuries the Irish possessed considerable knowledge of botany and the medicinal virtues of herbs and plants. [This was probably the period of the labours of the Arabistæ.] Boate and Sir William Petty followed in the seventeenth century; the former added in his works the knowledge of his brother, then physician-general to the English forces in this country. In the seventeenth and beginning of the eighteenth centuries some distinguished Irish scholars, Threkeld and Molyneaux in particular, availed themselves of the popular knowledge of the Irish people with regard to the medicinal virtues of plants; and the former states that he was much indebted to the Irish MSS. for information on that subject. These writers were followed by K'Eogh, but he added little to the labours of his predecessors; and the principal materials of his work on botany and the medicinal virtues of plants are evidently derived from those of Threkeld, Boate, and Molyneaux. These were followed by O'Connell and Rogers, of Cork, and Drs. John Rutty, Bryan Robinson, Garret Hussey, and others, from the beginning to the middle of the eighteenth century." (Report upon the Tables of Death, pp. iii-iv.)

Of the Irish medical worthies of the eighteenth century here mentioned, one, Dr. John Rutty, has always impressed us by the worth and, at the same time, singularity of his character. Besides having published many works on natural history and medicine much esteemed in their day, he kept a diary, less of what he did than of what he thought and felt, during the last twenty-two years of his life, from 1753 to 1774. This diary was published after his death. It presents an example of the most stringent self-catechising ever witnessed, generally expressed in a tone so quaint and humorous as strongly to arrest attention. It has been quoted and praised by that distinguished lover of all curious reading, Sir Walter Scott. The author often accuses himself of having eaten "swinishly" or "piggishly." On other occasions he "was sinfully dogged and snappish." One complaint he makes with which the profession will be sure to sympathize: "Eight patients and never a penny." His love of natural knowledge he regards, if not with self-censure, certainly with jealousy: "Moderation in my calling is witnessed; may it be extended to my two darling objects, natural history and the materia medica." From some passages in the Diary the reader might be led to think the author "a self-tormenting sophist;" such, however, is not his character, as displayed by the general tenor of the work. This reflects the image of a man of great benevolence; one aiming incessantly at the improvement of his moral nature, and, in every misfortune that may overtake him, having ever a ground of consolation at hand. "I have laboured," says he, "considerably, but not suffered much: perhaps a little in the withholding of fees *for telling truth ;* but what then! this is only ridding of superfluous wealth, an incentive of luxury."

The classification adopted by Mr. Wilde in his statistical nosology is, with slight variations, the same as that of Mr. Farr in his Reports to the Registrar-general. Mr. Wilde's nosology consists of four columns. In the first are given the terms used in the abstracts; in the second, the synonymes, analogous diseases, popular and provincial terms; in the third, the Irish names; and in the fourth, the English translations of these names. The Irish names appear from the translation, our sole means of judging, to be very expressive, and, in some examples, boldly figurative. For instance, we have prolapsus uteri; Irish name, tuitim an mhaclaigh; translation, falling of the place of the son. The causes of death are arranged under ninety-four heads. To some of these we shall now advert, and shall select the twelfth head, fever—a subject of much interest in Ireland and elsewhere.

The maculated or spotted fever, the true *typhus Hibernicus,* is recorded in the early Irish MSS. under the term of fiabrhus morgaighthe, or the putrid fever; and also fiabrhus righim, the lingering or low nervous fever. It is probable that this disease has always been endemic in Ireland; for Gerald Boate, in the seventeenth century, classes it among Ireland's *morbi endemii* under the name of the Irish ague, which all authorities now agree was the synochial and typhus fever. But the first defined period of epidemic fever in Ireland is that chronicled by Rogers in Cork and its vicinity, in 1708, and again 1718-21, and 1728 to 1731. These dates accord with the estimate of Mr. Wilde, that fever has raged nearly decennially in Ireland for the last 150 years. He does not intend to speak precisely; but would say, as a general rule, that fever has become epidemic from the eighth to the twelfth year, with an interval of from six to eight years; and facts during the eighteenth century establish his case very well, with the exception of two lapsed periods. Thus there was an epidemic in 1708, one in 1718-21, 1728-31, 1740-43, 1763-64, 1771-73, and 1817-21. The lapsed periods—that from 1743

to 1763, and that of thirty-four years, from 1773 to 1817—he seems to think may arise from deficiency of records for the period; admitting, however, that the accustomed visitations did fail in the periods mentioned, yet is the recurrence nearly at the decennial period sufficiently marked during much the greater part of an entire century to be, as he expresses it, curious and unaccountable; and we fully concur in the striking analogy he discovers between the course of epidemics and another natural phenomenon, as depicted in the language of Scripture: "The wind bloweth where it listeth, and thou hearest the sound thereof, but canst not tell whence it cometh and whither it goeth."

Though acknowledging fever to be the plague of Ireland, and from the period of historical record the most prevalent and fatal (epidemic) affection to which the country has been subject, Mr. Wilde very properly guards his readers from exaggerations founded on rough guesses. In the most fearful epidemic of the last century, that of 1740 and 1741, which was recorded by Rutty and O'Connell, the latter writer states the number that perished at 80,000; whilst the accurate Rutty says it was computed, *though probably with exaggeration,* that one fifth of the inhabitants died of fever. Again, in more recent times it has been asserted before a select committee of the House of Commons (in 1830,) that 65,000 died of fever in 1817. What reliance, asks Mr. Wilde, can be placed in such guesses, when it is discovered by statistics derived from sources much more accurate than former days possessed, that the mortality from fever was in ten years little more than 112,000, and cholera, in its three years' progress, carried off little more than 45,000.

One of the most appalling epidemics that ever invaded Ireland took place in 1817-18-19, and had Drs. Barker and Cheyne for its historians. In their report it was stated, that, "assuming the population of Ireland to amount to six millions, it will be no exaggeration to state, that a million and a half of persons suffered from an attack in the time included *between the commencement of the years* 1818 *and* 1819. In the course of the two years commencing with September 1817, more than 42,000 persons were admitted into the hospitals." During this epidemic the total number of persons admitted into the hospitals of Ireland (both temporary and permanent) was 100,737, of whom 4349 died.

This we consider a small mortality relatively to the number attacked; being but 1 in 23. The mortality was greater among males than females, in the proportion of about 60 to 51. In the mortality from fever during the decennial period embraced in the present report, (from June 1831 to June 1841,) it is somewhat curious to observe that the same proportionate preponderance of male over female deaths is observed, the proportion being 100 males to 86·14 females, or, within a very insignificant fraction indeed, the same as in the former case. During the ten years comprised in the report, the deaths from fever amounted to 112,672; and there was an epidemic of the disease of a malignant nature as an accompaniment to the influenza of 1836-1837.

Consumption, Mr. Wilde says, is by far the most fatal affection to which the inhabitants of Ireland are subject, exceeding the returns from fever by 23,518 deaths during the ten years. It is reported to have destroyed 135,590 of the population of those families from whom the returns were received on the 6th of June, 1841,—being to the deaths from all causes 1 in 8·75; to those of the entire class of diseases of the respiratory and circulating organs 1 in 1·31; to the total number of deaths from epidemic, endemic, and contagious diseases, as 100 to 281·17; and to fever alone as 100 to 82·65. The sexes are 100 males to 113·07 females, being the only one of this class of diseases, except asthma, where the females preponderate. The mortality from this melancholy cause appears from the returns to be very steady, for in 1836, the deaths were 14,603; in 1837, 16,078; in 1838, 15,481; in 1839, 16,673; in 1840, 16,126, and in the first half of 1841, 8201. He says:

"*A vast multitude of deaths* occurring in extreme infancy, and during the very early periods of life, were returned as having been caused by consumption, decline and decay (the two latter being the most common terms for this disease among the lower orders); now, although it cannot be denied that tubercular phthisis may occur at any age, (instances are recorded of tuberculous matter, and even cavities, being found in the lungs of new-born infants); yet, as it is not a general disease of very early life in this country, it became necessary, in arranging the registration, to limit the age at which deaths were to be registered as having been caused by phthisis pulmonalis. That age has been made the 10th year, and all cases returned of an earlier age have been placed under a separate head, that of marasmus, No. 50 (which see, page xxxii), for the reasons there detailed, as being the best term under which to register cases of the tubercular disease of early life (*phthisis infantum.*") (Report on the Tables of Deaths, p. xxviii.)

When we turn to the section "Marasmus" referred to, we learn that the term is used as a generic name, under which to class all those various affections of infancy and early youth returned in the census papers as consumption (infantile), wasting, decay, decline, emaciation, general debility, loss of strength, &c., and not intended to apply to *tabes mesenterica*, or any other state of atrophy in particular. This arrangement became necessary from the number of deaths returned as *consumption* and *decline*, under 1 year of age, and from 1 to 10. The total deaths registered under *marasmus* amount to 68,650, or about one half the number returned under the head of phthisis pulmonalis; the sexes being 100 males to 105·81 females, as in the case of *phthisis*, the latter sex preponderating.

From what we observe in England, where genuine phthisis is by no means infrequent in mere infancy, and of very considerable frequency indeed under 10 years of age, we cannot help believing that a very considerable proportion of the mortality classed under the head of *marasmus* ought to have found a place under *phthisis*. We admit that from the mode in which the materials for the tables of mortality were collected, it would be impossible to define the precise proportion in which cases should have been referred to the head of phthisis; but we have a conviction that to have been accurate, it must have been considerable; and this conviction adds to our impression of the great prevalence of consumption in Ireland.

The Irish name of phthisis is very significant and expressive, *seilgean as*—a shrinking of one's self. *Cnai*, or *cnasidh*, (we wish Mr. Wilde could help us to the pronunciation of these names,) a wasting or decay with or without disease of the chest, is the Irish term for marasmus. When the disease presents itself in the form of *tabes mesenterica* or general atrophy, many superstitious notions are entertained by the peasantry respecting its probable cause, such as the child being under the influence of spirits or fairies, the "gentry," or the "good people;" and hence the term in such common use, "back-gone," or "fairy-stricken," more than one of the A. forms gave these returns, and one, "the fairies tuck him away." These superstitions, though not unpoetical, yet naturally suggest an inquiry into the amount of education-grants to Ireland.

Under the 87th head, or that of intemperance, we find historical information of much interest, and in one respect not unpleasing, for it shows that this disgusting and destructive vice is on the decline. The deaths in the register ascribed to it immediately or remotely amounts to 1239 or 1043 males, and 196 females. The evidence of the existence of this evil during at least two centuries abounds. Stanihurst and Dymmok allude to the practice during the 15th and 16th centuries. In 1724 the pernicious use of ardent spirits became so manifest in the city of London, that the College of Physicians there put forward a public manifesto detailing its calamitous consequences. "It is also to be observed," says the worthy Rutty, in his Natural History of the County of Dublin, "that with us in Dublin the use of spirituous liquors, nearly from the same fatal era (1724), began to prevail, and from thence to the present time hath gradually prevailed to an enormous degree." After expressing what appears to us rather a whimsical speculation than a sound conclusion,—though he asserts that it is founded on the baptisms in Dublin, for 41 years,—that the drinking of ardent spirits unfits men for the propagation of male progeny, though leaving them the power of producing females, the same authority informs us, that Sir William Petty (in the 17th century) estimated the number of alehouses in Ireland at one third of houses generally. Dr. Rutty gives the following enumeration of houses of this description in Dublin in 1749: of alehouses, 2000; taverns 300; brandy-shops, 1200;—total, 3500, being nearly Sir W. Petty's proportion for the whole country. The following are Mr. Wilde's sensible, and on the whole encouraging remarks on this subject:

"This baneful thirst for intoxicating drinks appears to have gone on increasing to an extent that can hardly be credited (and will, it is to be hoped, be scarcely believed in future ages), from the days of Rutty to almost the present time; for when we sum together the deaths caused directly by the excessive use of spirits—the 77 deaths enumerated under the head of *delirium tremens*—the number of cases of lunacy that were induced by the same evil—and the vast multitude of accidents that have been the result of drunkenness,—then, I think, the loss of life caused by the use of ardent spirits, within the 10 years, cannot fall short of 2000, independent of the number of diseases induced, or rendered fatal, by its previous injurious effects upon the constitution, and the train of want, sickness, misery, and destitution, entailed upon the wretched families of those persons who were its unhappy victims. From the records furnished by the census inquiry, it appears that this description of death increased rapidly from the year 1831 to 1839. Happily, a new and better state of things has commenced, the effects of which had already begun to tell, in the two last years over which this inquiry extends; and, compared with the days of Rutty (1772), our improvement is evident, for, by the Dublin Police Report for 1841, it appears that there were only 880 public-houses and taverns, and 112 grocers who retail spirits by permission of the customs, which, with 106 unlicensed houses and 'grocers' shops, that 'retail spirits without a license,' amounting in all to but 1098, presents, in proportion to the population of the metropolis at the present day, an astonishing decrease in places of this description, since 1749. Including the cases of *delirium tremens* with the deaths from intemperance, during the 10 years ending 6th June, 1841, it affords a proportion of 1 in 33·77 of all the violent and sudden deaths, and 1 in 902·25 of the general mortality of the kingdom from all causes." (Report on the Tables of Deaths, p. xli.)

In connexion with this subject, we are surprised not to see the immortal name of Father Matthew: but we suppose the dignity of an official report did not permit this.

On the subject of the comparative mortality of England and Ireland, Mr. Wilde presents some general remarks, which appear, to say the least, plausible, though founded rather on speculation than sound statistical grounds. Considering the larger proportion of the rural compared with the civic district in Ireland than in England, and the much greater comparative mortality in the population of the latter than the former, ranging, for instance, from 1 to 64 in the agricultural districts and the open country, to 1 in 28 in densely-populated manufacturing towns, such as Manchester, &c.,—he infers that the rate of mortality in Ireland must be less than in England. The annual proportion of deaths to the living in the latter country is, on the average of the period during which the registration act has been in force, 1 in 45; whilst, from an examination of the entire records of disease, death, and longevity, in Ireland, Mr. Wilde is led to believe that were an accurate registration effected, the *proportionate* annual mortality would not be found to exceed 1 in 52, or 1 in 53. All this sort of reasoning sounds, we admit, very plausibly; but we cannot help being impressed with the very different conclusion reached by the commissioners, who, in words which we have already quoted, state that there can be "no reason for supposing the mortality less than 1 to 45, on the contrary, many circumstances would appear to place it higher."

Founded on a table of proportionate mortality in each county and province of Ireland, he classes the counties in the following order: In the first class, which includes those of Tyrone, Down, Fermanagh, Londonderry, Donegal, Armagh, Antrim, Carlow, Kilkenny, and Sligo, the average proportionate mortality, or ratio of deaths to the living, in one year, is 1 in 62. In the second class, including the counties of Limerick, Mayo, Westmeath, Kildare, Kelly, Cork, Longford, Queen's, King's, Wexford, Wicklow, Cavan, Monaghan, Waterford, and Leitrim, the average is 1 in 57·5. And in the third class, including the counties of Clare, Dublin, Louth, Meath, Galway, Tipperary, and Roscommon, 1 in 51·7. In these calculations, the specified cities are not included; their mortality, with the exception of Galway, offers a remarkable similarity, varying only from 1 to 32·12 in Dublin, to 1 in 35·05 in Belfast.

There is a table of deaths in towns, (exclusive of the cities of Dublin, Kilkenny, Cork, Limerick, and Waterford, and the towns of Drogheda, Belfast, Carrickfergus, and Galway,) which, could the *data* on which it is founded be implicitly relied upon, shows admirably the effect of density of population on sickness and mortality. In towns of 2000 population, and not exceeding 5000, the mortality from all diseases amounted to 1 in 50·85; in towns above 5000, and not exceeding 10,000 to 1 in 45·24; and in towns above 10,000 to 1 in 43·08; whilst in the open country the deaths were 1 in 76·93. With the exception of a singular deviation in the case of smallpox, in the case of which disease the mortality in towns of the first class, or those of the smallest population, was less than in the open country, the deaths from special diseases observed the same ratio as the total deaths, the mortality in towns increasing in proportion to the population, and being very much less in the open country. We give fever as an example. The deaths from this cause in towns of the first class, were 1 in 532·4 ; in the second, 1 in 439·2 ; in the third, 1 in 366·2; and in the open country, 1 in 864·3. Nothing can manifest more strikingly the effect of increased density of population in increasing the rate of mortality; and even admitting, as the commissioners do, that there are errors in the numeration ; yet, as it is presumable that these errors would be pretty nearly equal in the different localities, the table still retains its value.

Mr. Wilde furnishes a very lucid commentary on the "general table of deaths by years and ages," which is found at page 163 of the "Summary of Ireland." There took place, however, within the ten years 36·120 deaths of which the ages were not specified. But of 1,151,254 deaths, of which the ages were described, 269,199 took place during the first year of life ; 435,117 during the first five years ; and one half up to the 20th year or between it and the 21st. This he would regard as fixing the average age, which he arrives at by finding the mean period of a given number of deaths. For example, if, in 11,480 deaths in the county of Carlow, it be found that one half of the entire die under, or at the 29th year, then that becomes the average age or mean point at which death took place, and not, as in other statistical returns, by summing the ages and dividing by the number of deaths. Certain of the results thus obtained are certainly very extraordinary; for instance, the average age ranges from 6 in Drogheda, and 6 to 7 in Galway, two of the most unhealthy towns in Ireland, to 31 in Wexford county, the rural district of which, it has long been a matter of history, is one of the most healthy. We would remark that a table of the ages at which the total deaths (1,187,374) took place, which will be found at page xlvii of his Report, furnishes evidence of great longevity. We find, for instance, 25,877 deaths, male and female, at the age of 80 ; 3958 at 90 ; and 2445 at 100. It should be observed, certainly, that these are marked at the decennial period, when, as Mr. Wilde remarks, there is a predominance of deaths returned, as being of that age beyond the reality.

We now return to a subject of great moment, as well in this country as in Ireland—coroners' inquests. After much delay Mr. Wilde succeeded in obtaining a return of 20,334 deaths upon which coroners' inquests had been held. These deaths he arranges into six classes. Of these, the first comprises deaths caused by violence, neglect, evil intent, or design, of some second or third party, in short, homicides; the second, suicides; the third "accidental deaths, caused without intent or design;" the fourth, "deaths from natural causes," consisting chiefly of those taking place in gaols and prisons, or those occurring so suddenly, or under such suspicious circumstances as elicited judicial investigation; the fifth, deaths from the immoderate use of ardent spirits; and the sixth, those cases in which the cause of death was uncertain.

Of the eight specified varieties of death, included under the first class, those by murder amounted to 890, the majority of which took place in the rural districts. It is pleasing to observe that the returns show a progressive diminution of this horrible crime. During the first five years, from June 1831 to June 1836, the annual average number of deaths from this cause was 1008, and in the last five years 722, while in the last quarter period the two years and a half, ending the 6th of June, 1841, the number had decreased to 576 (annually). The proportion of males to females, for the whole period, was 47 to 1; but this number was seriously augmented by the marked preponderance of females in 1837, over all the other years; and this remark holds good with regard to each of the provinces. The months in which most murders occurred were December, January, March, and June, while April, July, and September were the least. Compared with the existing population the proportion of murders throughout the provinces was, in Ulster, 1 in 24,858; Connaught, 1 in 8496; Leinster, 1 in 8223; and Munster, 1 in 6191. This comparison is in the highest degree favorable to the north of Ireland. Mr. Wilde mentions the very curious fact, that of 119 homicides in the county and town of Galway, but 5 were returned as deaths by manslaughter; out of 434 homicides in the county of Tipperary, 333 were pronounced by coroners' juries to be manslaughter. Was the difference in the nature of the offences, or in the minds of the juries?

The inquest returns have specified six particular modes of death under the head of suicides; viz , those by hanging, drowning, gun-shot wounds, stabbing and cutting, poisons, and suffocation, which, with those where the means of destruction have not been specified, amount to 755 in all; 474 males, and 281 females. Nearly a third of these deaths were caused by hanging; about a fifth by drowning; less than one tenth by stabbing and cutting; somewhat more than a twelfth by poison; more than a seventeenth by gun-shot wounds; and scarcely one thirty-seventh by suffocation. The proportion of the sexes varies according to the means of destruction employed; thus hanging, stabbing, and cutting, and suffocation, were the means chiefly employed by the males, while drowning and poison were those principally made use of by females; fire-arms were in no instance resorted to by females.

Inquests were held upon 8072 cases (6083 males and 1989 females) of accidental deaths, caused without intent or design. The causes of death returned were: drowning, 2410; killed by wheel-carriages, 603; burns and scalds, 532; cold and exposure, 495; suffocation, 369; killed by animals, 214; falls from horses, 200; killed by the falling of masonry, 118; gun-shot wounds, 79; poison, 68; suffocation, from the injurious effects of lime-kilns, 43; killed by machinery, 40; by the explosion of gunpowder, 33; by lightning, 28; *starvation*, 25; and from eating improper food, 4. The deaths from unspecified causes amount to 2811.

In the fourth class, or deaths from natural causes, there are 5417 cases (3774 males, and 1643 females), in 4156 of which (a very large, and, we would say, censurable portion), the immediate cause of death was not specified, the verdict generally recorded by the jury being, "Died by the visitation of God." The specified causes amount to 14, viz. apoplexy and paralysis, 421; still-born, 315; hemorrhage, 112; epilepsy, 108; diseases of the chest, 75; age and debility, 72; fever, 46; insanity, 39; diseases of the cavity of the abdomen, 24; childbed, 17; mortification, 16; cholera, 9; concussion of the brain, 6; and cancer, 1.

The deaths from the immediate use of ardent spirits amount to 1129; that is, 950 males, and 179 females. The inquests upon these deaths divide themselves into, intoxication, 902, in which death took place either immediately, either upon drinking immediately of whiskey, or while persons yet laboured under intoxication; and 225 deaths caused by the united influence of intoxication, cold, exposure, or suffocation.

In the sixth class, "cause of death uncertain," including all those cases in which a verdict of "found drowned," "found dead," or, "the cause of death not known," has been returned by the juries, the whole amount to 1407; that is, 991 males and 476 females.

At the conclusion of this portion of Mr. Wilde's report, we have what we regard as very important information conveyed to us in the following statement:

"In 12,762 instances in the total number of inquests, medical evidence was had recourse to in the different classes, according to the following proportions: 1st class, 10 with medical evidence to 1 without; 2d class, 10 to 6; 3d class, 10 to 8; 4th class, 10 to 5; 5th class, 10 to 5; 6th class, 10 to 9. In 315 inquests held upon new-born infants the coroners' juries determined, *without the examination of medical evidence, that* 99 *were still-born;* while in 79 instances of accidental gun-shot wounds, medical evidence was had recourse to in 60 cases!" (Report on the Tables of Death, p. 41.)

Let us, on this side of the Channel, beware how we exclaim, "See, how prettily matters are managed in Ireland!" till we have set our own house in order. We feel a conviction, from what is passing under our own eyes, that were a return obtained, from the provinces of England, of coroners' inquests, specifying those in which medical evidence was resorted to, and those in which it was omitted, the proportion of cases in the latter predicament would be found larger in this country than in the "Green Isle." Such a return could readily be obtained by the home secretary, for his own information, and that of the registrar-general. Were the return so framed as to comprise in every case the verdict, whether medical evidence had been resorted to, and whether this was founded on anatomical examination, the latter authority would speedily discover what a mockery and delusion constituted a portion of his annual reports. It would be generally discovered, too, that verdicts, however they may have the "outward and visible signs" of precision, however scrupulously they may eschew the old and hackneyed phrase, "Died by the visitation of God," are yet mere guesses, in which the chances are at least twenty to one against the guessers, for want of that medical evidence which is so carefully and habitually excluded; John Bull would discover, too, that there was "a solemn farce" daily performed all over the country, which ended in a tragedy, in that very sensitive part of his system—the purse.

It would be out of place to burthen our pages with facts in support of our allegations. The return we have suggested would at once show whether they are well founded. Besides, facts have been already published, in an appropriate quarter,* showing that, in certain parts of the country, matters are conducted as we have represented them to be.

* See on this subject, in the Provincial Medical and Surgical Journal, No. 175, a letter from Mr. Lane of Grosmont.

How, it may be asked, has such a state of things been brought about? Why is it that that medical evidence, which the interests of medical science, and the community, and the efficient working of the registration act equally demand, is thus scrupulously excluded? We shall endeavour to explain this. Not as a consequence of any new legislative enactment; but mainly as a difference in the practical working of the existing law, the number of inquests holden throughout the country has very much increased. This is in part owing certainly to increased population and the growth of machinery; but is principally attributable to the circumstance that parish-constables and coroners give a more extended signification to the law than it previously possessed. An institution, manifestly framed solely for the detection of crime, as is evident, from the terms of the law on the subject, which expresses that the coroner is to inquire into cases of *unnatural* and *violent* death, is strained to embrace all cases of sudden death, and all cases regarding which any obscurity exists as to the cause, however exempt may be the deaths of both classes from all suspicion of homicide or suicide. If we add to this that the law is stretched so as to include, under the head of violent deaths, many cases not resulting immediately, but mediately or remotely, from violence, such as deaths from *tetanus*, and deaths, which only by a forced construction of the word can be considered violent at all, such as those from *delirium tremens*, one need not feel surprised at the increased bustle in coroners' courts. But if the bustle be great, the cost is great too, and great is the complaint and remonstrance of magistrates, in quarter-sessions assembled, at the inflammation of the county rates by coroners' charges. But these latter gentry are not absolutely indifferent to the frowning aspect of that great abstraction—"Quarter sessions;" nay, so far from being indifferent, they quail before it; and, being unwilling to propitiate this awful "Cerberus" by a diminution of the number of inquests and their own fees, they endeavour to curtail the expenses of each inquest by the exclusion of the only evidence which can give their verdicts any value, medical evidence. The "Medical Witnesses Act," which gave a guinea, and in case of anatomical examination of the body, two guineas to each medical witness, was devised, we are convinced, in a friendly spirit to the profession, and with the intention of securing evidence deserving of credit, for the detection of crime, and for the illustration of obscure deaths; but the practical operation of this act, in the provinces of England at least, has been diametrically opposite to that which Mr. Wakley intended.

The question will naturally arise, how is this state of matters to be corrected? We candidly admit that we have no hope from any modification of coroners' courts. We cannot conceive the possibility of modifying an institution, framed in a barbarous age, when homicides were abundant, purely for the detection of homicide, and for the assessment of the damages due from the hundred where the crime was committed, so as to make it a fit instrument for the discovery of the cause of obscure death in the present day. A Bill is at present (March 1844) in progress through Parliament, and will certainly pass, which invests magistrates, in quarter sessions, with the power of increasing the number of coroners' districts, and thus, by augmenting the number of these functionaries, and bringing each one nearer the work he has to perform, of diminishing one item of cost, the coroners' travelling expenses, (technically called mileage ;) but lest this should, by possibility, be attended with any beneficial result, and diminish the murmurs of quarter sessions, by this very bill the allowance of mileage is, with unaccountable absurdity, increased from ninepence to one shilling per mile. We need not say that no good can possibly arise from such legislation as this.

But we believe that even skilful legislation would be wasted on machines so ill adapted for the purpose now required as are coroners' courts. No Coroners' Amendment Act can answer any good object. Thirteen persons, often humble in station and education (in one case, as Mr. Lane* informs us, a stable-boy) are brought from their daily vocations, at much inconvenience to themselves, and are anxious to get the business over as speedily as possible ; and as soon as they discern that there is no reason to suspect homicide, they promptly agree to any verdict, to which they may be led by the coroner (generally an attorney), anxious to avoid any cost beyond the necessary expenses of the court, which are always abundant. Jurors will ever take the view, that, provided there is no homicide, their vocation is at an end ; and this limited view we have known them persist in taking, in spite of the remonstrances of medical men, when such have been present. Cases of sudden and obscure death, if duly investigated by a medical man, surely can receive no additional illustration from a coroner and his jury. Let such cases then, be decided by medical officers appointed in different districts of the empire, to discharge functions similar to those performed by the medical officer of the morgue, at Paris, and to these let sudden and obscure deaths be referred. Where suspicion of homicide exists, or where it may arise in the course of the medical officers' investigation, then let the case be examined by the (now) well-organized police and magistracy of the country, who have shown themselves, as we ourselves have witnessed, more skilful in the detection of crime than a parish constable and a coroner's court. Some exclamation may be heard touching that "Palladium" of English liberty,—a jury ; but let it be remembered that there are still two juries between one accused of homicide and conviction, a grand jury and a petty jury of assize. Let, then, an institution be abolished,—totally unsuitable to the requirements of the present day ; and which has become an obstacle almost insurmountable to the due examination of a class of deaths, which the interests of medical science, and those of the public require to be fully investigated.

We quit this digression, if that can be called a digression which has the most direct relevancy to what must be the foundation of all vital statistics, correct registration, to express the high opinion we entertain of this truly complete Report, *complete*, at least, so far as attainable sources of information rendered fulness and accuracy possible ; and to bestow well-merited commendation on the learning and professional knowledge displayed in Surgeon Wilde's Commentary on the Tables of Death.

* See his letter, already referred to, in the Provincial Medical and Surgical Journal.

